# In-Organization Ethics Power-Allocation Mechanisms and Members’ Decision-Making Behavior

**DOI:** 10.3390/bs12010006

**Published:** 2021-12-28

**Authors:** Yudan Pang, Xuefeng Wang, Hang Wu, Fanfan Zhang

**Affiliations:** School of Management, Harbin Institute of Technology, Harbin 150001, China; wangxuefeng1125@126.com (X.W.); hang.wu@hit.edu.cn (H.W.); 19b910043@stu.hit.edu.cn (F.Z.)

**Keywords:** power allocation mechanisms, dishonesty behavior, decision making, ultimatum game, dictator game, organization behavior

## Abstract

This study examines experimental evidence showing how ethics power allocation mechanisms affect an individual’s in-organization resource division and ethical behavior. We used two two-stage lab experiments to explore power seeking and usage; the experiments contained two stages of power contending and power usage. Stage one used two different power-seeking mechanisms in the honesty game. Stage two was based on the dictator game and the ultimatum game to measure an individual’s power usage. The results show that the decisions taken by power-holders could influence the optimization of collective resources, and power-holders who gain power with unethical methods could result in collective resource allocation inequities. With more balanced in-organization power, members tend to be more honest. Subjects also adjust their unethical behavior to adapt to the environment, which could cause the diffusion of unethical behavior. This paper re-designed the dictator game and the ultimatum game by adding an ethically vulnerable power acquisition mechanism. For organizations to prevent the disproportionate dispersion of resources and achieve more public benefits, it is meaningful for managers to create a proper in-organization ethical power allocation mechanism.

## 1. Introduction

The process of achieving positive organizational aims and increasing collective benefits requires efforts from both leaders and other members in an organization. However, in an organization, ethical scandals often accompany the process of fighting for higher power status, and the potential risks of gaining power through unethical methods have been noted [1,2]. This could negatively influence the collective benefits of in-organization resource optimization. It is beneficial to explore how different ethical power-allocation mechanisms impact the in-organization members’ behavior in resource allocation.

In organizations or groups, internal power is directly related to an individual’s ability to access resources in groups or organizations. Power-holders in organizations can make decisions regarding collective resource allocation and could be a key factor influencing the process of achieving optimized resources. Increased resource allocation efficiency and the positive use of internal power can encourage organization members to achieve pro-social goals, for example, by enhancing green organizational culture. Power-holders can enhance collective interests and build a harmonious organization atmosphere, while their selfish behavior promotes self-aggrandizement and results in a centralization of resources in the organization [3,4].

However, in-organization power inequities can negatively impact a group’s emotions, and the power-holders in these circumstances are typically considered responsible [5]. A traditional view is that a greater imbalance in power often implies a wider gap in individual resources [6]. The distinguished behavior of group members of different power or social statuses is also deeply influenced by their group or community’s ethical environments [7,8]. Under different power conditions, individuals can either be cooperative or competitive with regard to group welfare.

In this article, we experimentally studied the influence of different ethics power-allocation conditions on the division of collective resources using a two-stage laboratory experiment. We explored the relations between an individual’s unethical behavior to gain power and the resource allocation within organization members. We used two classic games in economics: the dictator game and the ultimatum game. In our experiments, we added an initial stage with two power-seeking mechanisms, dishonesty inducing and dishonesty proof, to assign roles to players and create a distinguished ethical environment for power seeking. In the dishonesty-inducing scenario, subjects can act dishonestly to acquire an advantage in power seeking. In the dishonesty-proof scenario, the roles are randomly assigned. Our 2 × 2 experimental design features four experimental treatments with different ethical environments and power conditions in order to study participants’ behavior in the power seeking and using stages.

We found significant deceptive behavior in the dishonesty-inducing scenario for subjects with both higher and lower power states. The multiple-round experimental results show that different power states between members can impact their dishonesty. Power-holders in a dishonesty-inducing scenario presented significantly lower intentions of behaving dishonestly in an organization with more balanced power states than unbalanced power states. The subjects tended to increase dishonesty degrees over time and adjust their intentions of behaving dishonestly after the results from previous rounds. This adjustment of a dishonesty-inducing environment can cause a diffusion of dishonesty among organization members. Our results also show that the power-holders in previous experiment rounds, especially those who behaved unethically, will have higher intentions of being dishonest to gain advantages in gaining power in the future. However, generous power-holders in previous rounds have relatively lower intentions of behaving dishonestly in future power-seeking procedures. When power-holders and other subjects have more balanced power states in the organization, the former will become more generous over time. Our research finds the negative impacts of dishonest behavior on a power-holder’s distribution of collective resources. The interactive impact of dishonest behavior and power-balance also indicates that when the power states between members are more balanced, the dishonest power-holders will increase their altruistic actions to prevent unwanted punishment, which could decrease collective resources.

The primary contribution of this article was the exploration of how different ethics power-allocation mechanisms affect individual behavior in both the acquisition and use of power. In addition to financial gains, the pursuit of power itself motivates individuals to engage in deception. We find that power gained through unethical methods typically results in larger profit dispersion and less altruistic behavior in groups. In contrast to the traditional negative stereotypes surrounding power and dictators, we find that different ethical power-allocation mechanisms are the key factors causing inequality between subjects in organizations. The unethical method of the power-allocation method in unbalanced power groups or organizations can decrease collective resources.

The remainder of this article is structured as follows. Section 2 reviews the relevant literature. Section 3 describes the research methods and experimental design and then presents the hypotheses; Section 4 presents and discusses the results of the experiments; Section 5 discusses potential extensions of the research and concludes.

## 2. Literature Review

Previous studies have highlighted the importance of members’ behavior for the benefits of ecosystem services programs [9,10], and experimental evidences have enhanced the idea that individuals’ pro-social behavior in groups or organizations could be great factors for ecosystem goods and be influenced by members inside or outside the organization [11,12]. Researchers have associated power and leadership with the dark side of human nature [1,13]. Inequities in power and wealth typically result in more fragmented social atmospheres [6]. Power-holders, or leaders in organizations are described as selfish, untrustworthy, corruptible and likely to abuse power [14,15]. Keltner et al. [15] showed that power status directly impacts individual decision-making, as higher power status implies greater social resources. Individuals with high moral awareness are less likely to engage in self-interested behavior [16]. Power inequities can result in changes in emotions and behavior. Negative inequity evokes envy toward power-holders, leading to damaging behaviors, while positive inequity causes guilt and motivates cooperation [5]. Moreover, changes in power status can have a significant impact on an individual’s personality and their performance in an organization [17]. Sometimes, self-interested behavior brings unintended outcomes. In a common-interest group, the power-holders’ self-interested behavior might also benefit group members of low-power status [18]. Members in organizations care about in-organization equalities by introducing a mixed dictator and ultimatum game experiments to increase proposer’s offer to others; the work by Chen et al. [19] indicates the importance of network reciprocity in enhancing the evolution of in-organization fairness.

Power-holders can have individual-focused and/or group-focused goals; however, achieving individual-focused goals makes power-holders cooperate less with individuals of low-power status [18,20,21]. Individuals focusing on group-focused goals can be less self-interested and motivate those with low-power status to work toward group goals [22]. Power-holders may even engage in self-sacrificial behavior to benefit group interest. However, group-focused goals may also drive some power-holders towards making unethical decisions that could benefit the group or organization [23].

Altruistic behavior can decrease the psychological costs of power-holders’ unethical actions [24]. Previous research has challenged the self-interested homo economicus hypothesis, showing that individuals tend to behave more fairly in dictator games [25]. Gneezy [26] found that individuals avoid deceiving others because of the psychological cost of unethical behavior. Individuals weigh the benefits of deception against the moral costs; those wishing to maintain a positive self-concept in society will prevent deception [27]. Another strand of literature found that the moral cost of harming others outweighs potential harm to oneself, as shown by the existence of altruism [28]. Power-holders tell big lies to achieve their personal interests but can reduce their psychological costs by telling minor lies [29]. Therefore, unethical behavior affected by power is not only undertaken by power-holders but also by others in the organization in order to obtain more resources.

However, lying does not necessarily result in moral costs. Moral awareness may vary as individuals achieve different goals; under some circumstances, individuals will consider benevolent lies to be more ethical than selfish honesty [30,31]. Organization members may engage in unethical pro-organizational behavior to increase collective interests and a power-holder’s personalities have nonlinear correlations with those unethical behaviors [32]. Self-regulation is also related to moral awareness. When individuals deplete their self-regulatory resources, their moral awareness will decrease, resulting in impulsive, unethical behavior [33].

A group’s ethical environment can influence members’ actions [8,34]. The process of establishing a group’s ethical environment and regulating individual behavior is interactive. In previous experiments, participants were more likely to engage in unethical behavior in competitive situations than in cooperative situations [35,36]. Zaki [37] shows that when individuals perceive changes in power status, their sense of empathy and perspective can decrease unethical intentions. When individuals violate group norms, other members begin to engage in unethical behavior as a method of self-protection. The chaos caused by declining moral awareness in a group results in a deteriorating ethical environment [38,39]. An individual’s behavior is affected by power and can, in turn, influence the group’s ethical environment. The interaction of a group’s ethical environment and members’ behavior will lead to behavior convergence.

Previous studies by economics and psychology experts using experimental methods have widely used the dictator game [40] and the ultimatum game [41] to explore individuals’ bargaining and charitable behaviors [42,43,44,45]. The two games have been used to examine individuals’ different actions through two proposing mechanisms, proposers and receivers [42]. Subjects in the two games of different power statuses have different behavior predictions. In the dictator game, previous research has predicted that proposers, to maximize their personal profit, should offer zero to the receiver, and face no risk of punishment for being selfish or benefit from being generous [44]. However, in the ultimatum game in which receivers hold veto power, the predictions indicate that proposers should offer the smallest possible non-zero value, and the receiver will accept any offer above zero. With the veto power, receivers will reject all zero offers as punishments [45]. Considering the natural design of these two games, greater inequities are seen in the dictator game. Korenok et al. [46] discussed the potential inequity aversion among dictators, and the results indicate that dictators’ offers to receivers are influenced by the inequity aversion and not all dictators follow the perfect Nash equilibrium in the dictator game. Cappelen et al. [47] and Besancenot et al. [48] explored the dishonesty behavior of individuals using the dictator and ultimatum games. The results indicate that the non-economic aspects of the choice situation are crucial in an individual’s dishonesty intentions. The researchers concluded that the two economic games are adequate tools for conducting explorations of an individual’s ethical behavior.

Previous literature has indicated that power has different impacts on individuals’ self-interested and altruistic behavior. It has also indicated the importance of equities of power and resources in organization members’ behavior. Although power inequities lead to negative outcomes, positive inequities may lead to collective benefits. Based on the above literature, we predicted that different power states will impact an individual’s ethical behavior. We then designed our experiment based on previous theories and designs to achieve our research aims.

## 3. Research Design

### 3.1. Experiment Design and Procedure

The experiments were designed as a 2 × 2 (dishonesty proof versus dishonesty inducing × dictator game versus ultimatum game) two-stage experiment. In Stage 1, the participants compete via a dice-rolling procedure for the power to allocate resources by moving first in the second stage. We adopted two power-seeking mechanisms: dishonesty proof and dishonesty inducing. In the dishonesty-proof group, participants who roll larger dice numbers N∈{0,1,…,10} in Stage 1 are assigned as proposers. In the dishonesty-inducing group, participants who report larger numbers N∈{0,1,…,10} in Stage 1 are assigned as proposers. Although the other subjects cannot see the actual numbers rolled on the dice in this dishonesty-inducing condition, we can observe and document the actual and self-reported numbers of all subjects through experiment software. Proposers are randomly selected in the case of ties.

In each treatment, two subjects set up an experiment unit and hold a collective initial asset of ω=30 experiment currency units (ECUs). Subjects with larger dice numbers can propose the allocation of initial assets in the next power-using stage. In Stage 2, subjects are assigned in either the dictator game or the ultimatum game treatment.

In the dictator game treatments [40], the first mover (the proposer) chooses an allocation scheme of the initial asset between themselves and the second mover (the receiver). We used ω, $p_{1}$ and $p_{2}$ to represent the initial money and monetary profits for the two subjects in this round, and g as the money given to the receiver. The two subjects receive $p_{1}=\omega -g\;\left(g\in \left[0,\;30\right]\right)$ if this subject acts as a proposer, $p_{2}=g\left(g\in \left[0,\;30\right]\right)$ if the subject acts as a proposer and $p_{2}=g\left(g\in \left[0,\;30\right]\right)$ if the subject acts as a receiver, according to the allocation scheme. The game then ends, giving the proposer greater power status among the two subjects. The Nash equilibrium in this game stage is that the proposer keeps all the money for maximum self-interest. Thus, if the subjects have maximum self-interest in this stage, then, g=0, $p_{1}=\omega_{\max}=30$, and $p_{2}=g_{\min}=0$.

In the ultimatum game [42] treatments, the first mover (the proposer) proposes an allocation scheme, which the second mover (the receiver) can either accept or reject. If the receiver accepts, the initial asset is divided as proposed, and two subjects receive $p_{1}=\omega -g\;\left(g\in \left[0,\;30\right]\right)$ if this subject acts as a proposer, and $p_{2}=g\left(g\in \left[0,\;30\right]\right)$ if this subject acts as a receiver; otherwise, both players receive nothing, and $p_{1}=p_{2}=0$. In the ultimatum game treatments, the receivers hold the ability to “punish” the proposers. The sub-game perfect Nash equilibrium for this ultimatum game is for the receiver to accept any positive offer and so for the proposer to propose the smallest possible amount. Thus, in this ultimatum game, if all subjects follow the Nash equilibrium, g=1, receivers will accept all positive proposals. The power status between two subjects is more balanced in this ultimatum game treatment compared with the dictator game treatments.

We conducted laboratory experiments from September to October 2019 at a Chinese university. We recruited 116 university students (56 males and 60 females) from a pool of registered volunteers; 52 subjects participated in the dictator game treatments (22 in the dishonesty-proof scenario and 30 in the dishonesty-inducing scenario), and 64 subjects participated in the ultimatum game treatments (34 in the dishonesty-proof scenario and 30 in the dishonesty-inducing scenario). The experiments were conducted on computers using z-Tree [49]. Before the experiments, the subjects were provided with instructions which the experimenter also read aloud. The participants played for 20 rounds with random re-matching after each round.

We used financial incentives for the participants in the experiments. In this experiment, 15 ECUs equaled CNY 1 (nearly EUR 0.14). All the experiments lasted for approximately 45–60 min, and the participants gained an average profit of CNY 35.26 (approximately EUR 5), including a CNY 10 (approximately EUR 1.4) attendance fee. This financial incentive matched the local hourly income. According to the post-experiment anonymous questionnaire, participants expressed high satisfaction regarding the monetary payoffs in this experiment.

### 3.2. Behavioral Predictions

We predicted that power-balance can lead to a decline in holistic profit in the whole group for compensation for unethical behavior. Power-holders will learn to avoid punishment by becoming more honest because low-power individuals, who value procedural justice, can punish power-holders. Considering the role of intentions, the kindness and ethics of other subjects’ actions will affect the subject’s decision-making [50,51]. The dishonesty-inducing environment may also lead to a fixed hierarchy of power and the power-holders may increase their dishonesty behavior to maintain power and resources. In a more balanced power environment, proposers will offer more to receivers to reduce inequity [52,53]. Moreover, we predict that individuals’ dishonest behavior and power-balance interact with and impact resource allocation. In a dishonesty-inducing environment, resource allocation in power-balanced groups is more equal.

We present the following hypotheses:

**Hypothesis** **1.** *Subjects in dishonesty-inducing treatments are more likely to behave dishonestly in the dictator game than in the ultimatum game because the power statuses among subjects is more balanced.*

**Hypothesis** **2.** *Subjects in dishonesty-inducing treatments have a greater degree of dishonesty in the dictator game than the ultimatum game. The deviations between self-reported and true numbers will be greater in dictator game treatments.*

**Hypothesis** **3.** *Power-holders will increase their future intentions of behaving dishonestly to maintain their power.*

**Hypothesis** **4.** *Generous power-holders having offered more balanced resource allocation schemes in previous periods will increase their honesty.*

**Hypothesis** **5.** *Power-holders will be more generous when receivers hold more power and will increase their offers over time. In the dishonesty-inducing environment, the power-balance will be an important factor influencing the resource allocation scheme.*

**Hypothesis** **6.** *Subjects’ dishonest behavior affects the offers to receivers. Power-holders who gain power with dishonest behavior will offer less to receivers. The greater the deception degree that power-holders have, the less generous they will be.*

**Hypothesis** **7.** *Subjects’ dishonesty behavior and power-balance have interactive effects on the resource allocation scheme. In the ultimatum game treatments, when there is less power inequity, the dishonest power-holders will offer more to receivers to decline moral costs and prevent rejections.*

**Hypothesis** **8.** *In the dishonesty-inducing treatments, receivers are more likely to accept the offers because they are larger in these treatments than in dishonesty-proof treatments. The resource allocation will be more equal in this condition.*

## 4. Results and Discussion

In this section, we present our findings in two primary sub-sections: (1) dishonest behavior from subjects in the power-allocation stage and (2) resource allocation behavior within subjects in the power-using stage.

### 4.1. Dishonest Behavior in Power-Allocation Stage

In this section, we present our findings in two main sections: dishonesty behavior from subjects, and resource allocation behavior in power usage.

We present the descriptive statistics in Table 1, including the average self-reported or true numbers in Stage 1, the average proposal numbers in Stage 2, and the percentage of acceptance of two ultimatum treatments. Additionally, the table presents the *p*-value from the *t*-test for the differences between the dishonesty-inducing and dishonesty-proof treatments. The results show the that the self-reported numbers in dishonesty-inducing treatments are both larger than dice numbers in the two dishonesty-proof treatments. The *p*-values also determine that the observed differences are statistically significant (*p* < 0.01).

**Figure 1:**
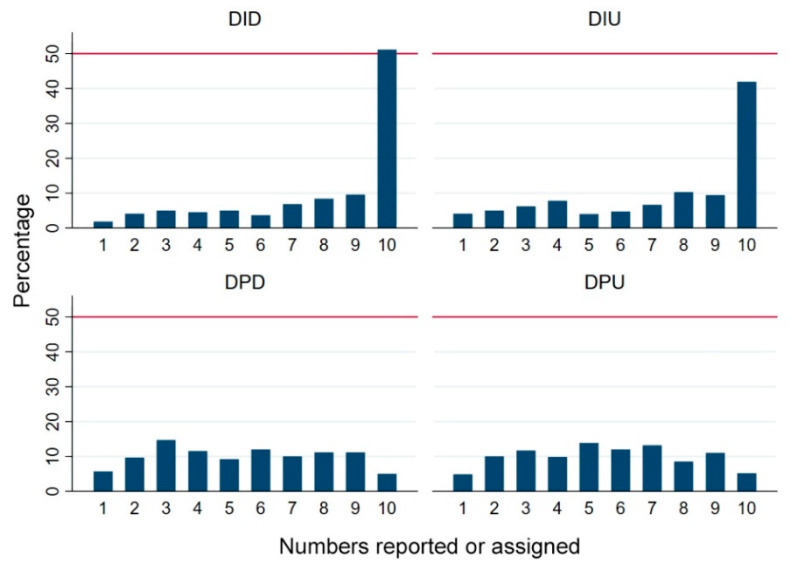
Percentage of self-reported or true dice numbers in four treatments. Note: The reference line represents 50%. DID represents the dishonesty-inducing dictator game; DIU represents the dishonesty-inducing ultimatum game; DPD represents the dishonesty-proof dictator game, and DPU represents the dishonesty-proof ultimatum game.

We present the percentage of subjects’ dice numbers (self-reported or true numbers) from four treatments in Figure 1. The percentage of larger numbers being reported is greater in the dishonesty-inducing dictator game. Subjects in the dishonesty-inducing treatments tend to be more dishonest when the power states are more balanced between subjects, and subjects prefer reporting larger dice numbers in the dictator game treatment compared with the ultimatum game treatment. When computed, the deviations between subjects’ self-reported dice numbers and true dice numbers ($\Delta_{N}=\left|N_{repo}-N_{true}\right|$) to measure the degree of dishonesty. The average $\Delta_{N}$ in the dictator treatment is 2.63 (*SD* = 3.05) and 2.17 (*SD* = 2.89) in the ultimatum treatment. The difference between $\Delta_{N}$ in the two treatments is statistically significant according to the Mann–Whitney *U*-test result (*z* = 2.77, *p* < 0.01). Subjects tend to tell greater lies to gain power when they report fake dice numbers in dictator game treatments.

Previous research has indicated that power can encourage deceptive behavior in organizations. We found that in a dictator game with greater power inequity, subjects present a higher degree of dishonest behavior. We also examined the probability of subjects being dishonest under different power-balance conditions. Table 2 presents the results from two Probit regression models on the subjects’ dishonesty behavior in two dishonesty-inducing treatments. The dependent variable dishonesty is a dummy variable, which takes the value 1 if the subject’s self-reported dice number is not the same as the true dice number. In model 1, the primary independent variables are: dummy variable ultimatum, which equals 1 if the treatment is an ultimatum game; period, which represents the round in each session; true number, which is the subject’s true dice number; age, which is the age of the subject; and the dummy variable gender, which equals 1 if the subject is a woman. We examined the effect of the results from the previous period on the subjects’ dishonesty. In a repeated experiment, the subjects may be myopic and concentrate on the current period or may adjust their behavior from the previous results. Therefore, in model 2, we excluded data from the first period and added three more independent variables compared with model 1: previous proposer, which equals 1 if the subject was the proposer in the previous period; previous offer, which is equal to the offer in the previous period; and previous deviations, which is equal to the deviations of two subjects’ self-reported or true numbers. These three independent variables can be used to explain the potential influences of the previous period’s results on subjects’ behavior during the later period.

The coefficients in model 1 indicate that the subject’s dishonest behavior is significantly affected by the type of game (*p* < 0.01). The ultimatum game type has a significant negative impact on subjects’ willingness to behave dishonestly. Period significantly positively impacts dishonesty (*p* < 0.01). Subjects will increase their intention of behaving dishonestly over time. A true number is a significant negative factor for subjects’ dishonest behavior (*p* < 0.01). Subjects who have already generated a larger dice number have a very low intention of behaving dishonestly. It can be deduced from the descriptive results presented above that subjects are more honest in ultimatum games. Subjects in the dishonesty-inducing ultimatum game have a lower intention of reporting fake numbers. Subjects have an increased probability of behaving dishonestly in the dishonesty-inducing dictator game, in which the power states are less balanced. From these results, we could deduce that the following.

**Result** **1.** *Subjects behave dishonestly in dishonesty-inducing treatments to gain power for Stage 2. In the dishonesty-inducing ultimatum treatment with more balanced power states between subjects, subjects behave more honestly than in the dishonesty-inducing dictator treatment. The balanced power states between subjects can efficiently reduce the dishonest behavior in groups.*

**Figure 2:**
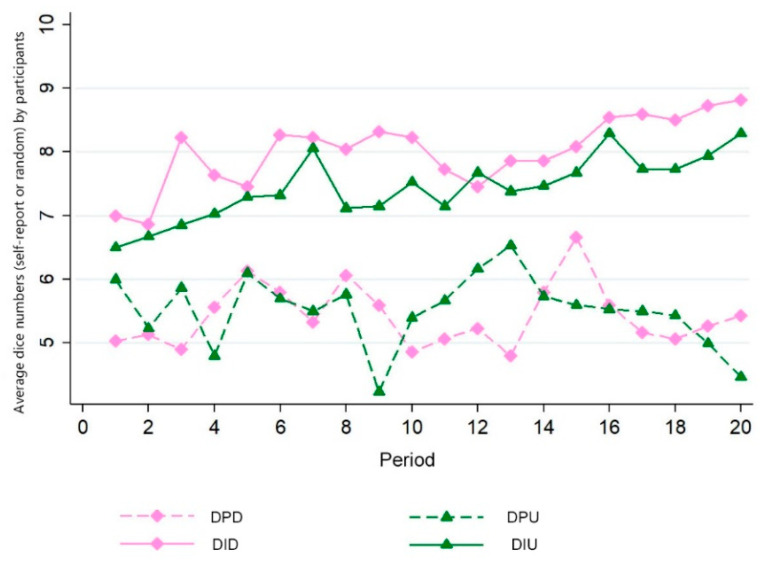
Changing trend of average numbers in four treatments.

In model 1, we found that in this repeated game experiment, subjects’ dishonesty is significantly affected during the period and that subjects can adjust their dishonesty over time. We present the changing trends of self-reported or true dice numbers over time in Figure 2. In this figure, the self-reported numbers in two dishonesty-inducing treatments increase with the period.

The results from model 1 and Figure 2 present a brief idea of how subjects’ dishonest behavior is affected by period. Based on model 2, we further discuss the potential impact of three independent variables from the previous period on subjects’ dishonesty. The independent variable previous proposer has a significant positive impact (*p* < 0.01). If the subject acted as a proposer in previous Stage 1, they would significantly increase dishonest behavior in the next period. The power gained from the previous period could affect subjects’ dishonesty. Subjects who hold more power beforehand are more likely to act dishonestly in the future to take advantage in order to gain more power. A previous offer has a significant negative effect (*p* < 0.01). When subjects have witnessed a larger offer in the previous period, they behave more honestly in the next period. The large offer to receivers is a fairer resource allocation plan and can decrease subjects’ sense of inequity in the resource allocation stage. Moreover, previous deviations can positively and significantly affect dishonesty (p < 0.01). From the results in model 2, we find that subjects will adjust their dishonesty and learn from the results from the previous period.

**Result** **2.** *In the dishonesty-inducing environment, subjects’ intentions of behaving dishonestly increases over time. Subjects will adjust their dishonest behavior over time to adapt to the environment, resulting in the diffusion of dishonesty among subjects. Previous power-holders will significantly increase their dishonesty intentions to take advantage in order to gain power in the future. Providing or receiving larger offers can significantly increase subjects’ future honesty. Larger previous deviations between two subjects’ numbers can increase subjects’ future dishonesty.*

### 4.2. Resource Allocation Behavior in Power-Using Stage

**Figure 3:**
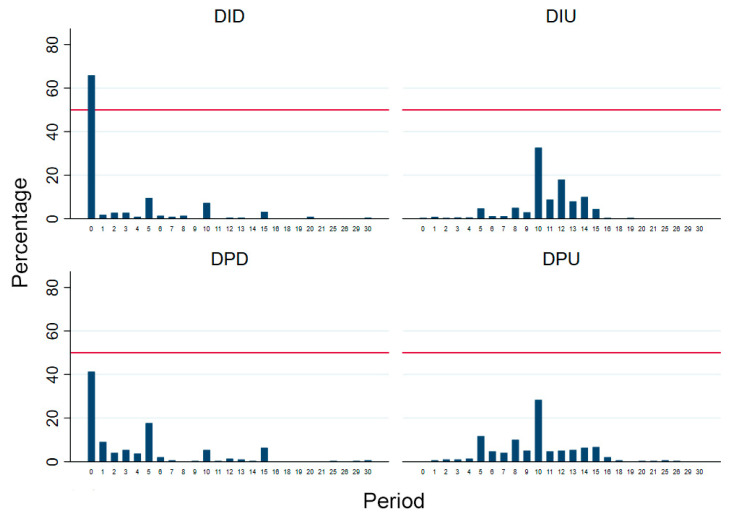
Percentage of offer values in four treatments. Note: The reference line represents 50%. DID represents the dishonesty-inducing dictator game; DIU represents the dishonesty-inducing ultimatum game; DPD represents the dishonesty-proof dictator game; and DPU represents the dishonesty-proof ultimatum game.

In Table 1, the descriptive results show that subjects display distinguished behavior in the resource allocation processes in Stage 2. The power between proposers and receivers is more balanced in the ultimatum game treatments, and proposers provide more money to receivers when receivers hold veto power. We present the percentage of proposers’ offers to receivers in the four treatments in Figure 3. Since the Nash equilibrium of the two game differs, we discuss the percentages of different offers separately. We compared the offers between dishonesty-inducing and dishonesty-proof treatments in the dictator and ultimatum games. Proposers in the ultimatum game offered more in dishonesty-inducing treatments than in dishonesty-proof treatments. However, in the dictator game, proposers in dishonesty-proof treatments offered more than in dishonesty-inducing treatments. We can observe in Figure 3 that, in the two dictator game treatments, proposers have a high probability of offering nothing. The rank sum test results also confirm that the differences between the four treatments are statistically significant (*p* < 0.01). Then, we conducted further analyses to explore the potential factors that could result in the differences in resource allocation behavior between treatments.

**Figure 4:**
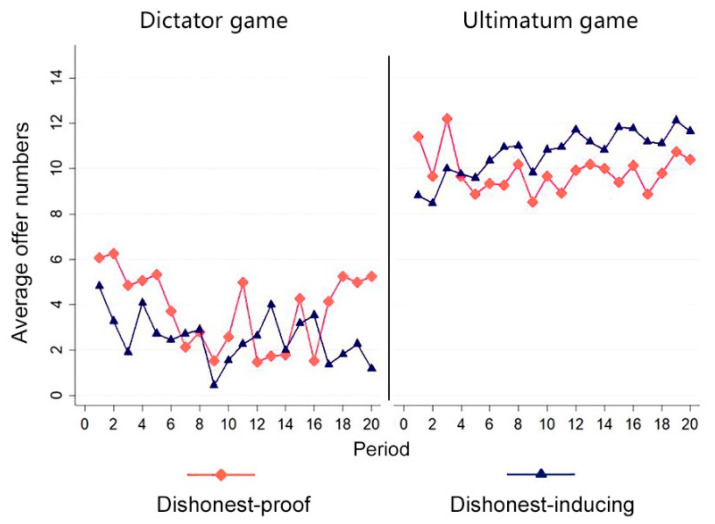
Changing trends of offers over time.

The offer value is directly related to the subject’s profit. We conduct rank sum tests of proposers’ profits in each period to examine the differences between four treatments. The results in Figure 4 indicate significant differences between them (*p* < 0.01). Proposers in dishonesty-inducing dictator treatment gained the highest profits in their offer period (Mean = 27.44, *SD* = 4.67) and proposers in the dishonesty-inducing ultimatum game gained the lowest profits in their offer period (mean = 13.92, *SD* = 8.19). We calculated the rates of acceptance in the two ultimatum treatments and found no differences; both were 75%. Then, the Mann–Whitney *U*-tests of acceptance between dishonesty-inducing and dishonesty-proof ultimatum treatments showed no significant difference of acceptance behavior (*z* = 0.016, *p* = 0.987). The receiver’s rejection actions are not the sole factor resulting in the differences between the subject’s profits. We conducted Tobit regressions on the offers provided by proposers to explore other potential factors. Table 3 presents the Tobit regression results.

In the Tobit regression models, we focus on the factors that may result in distinguished resource allocation behavior on the behalf of power-holders: the power-balance among subjects, dishonesty inducing or dishonesty proof, period, dishonest behavior, and inequity of self-reported numbers. The dependent variables are the offers provided by proposers.

In models 1 and 2, we included all treatments and examined the impact of the power-balance between subjects and two power-seeking mechanisms indicating the different ethical environments in organizations. The primary independent variables in these two models are the dummy variable dishonesty inducing, which is equal to 1 if the treatment is dishonesty inducing; ultimatum, which is equal to 1 if the treatment is an ultimatum game; period; self-reported numbers; gender, which is equal to 1 if this subject is female; and age. We also introduced a two-way interaction term in model 2, dishonesty inducing × ultimatum. From the regression models, we can see that the ultimatum game significantly positively impacts the offers from proposers (*p* < 0.01). Proposers provide significantly larger offers to receivers in the ultimatum games (*p* < 0.01). Proposers in a more balanced power status offer more. Dishonesty inducing significantly negatively impacts offers, and the interaction dishonesty inducing × ultimatum has significant positive influence (*p* < 0.01). In the dishonesty-inducing ultimatum treatment, proposers tend to be more generous and provide larger offers.

**Result** **3.** *Power-balance can benefit subjects in groups overall. Proposers are more generous over time when receivers hold more power, and the equities of power between individuals can in turn reduce profit inequities. In a dishonesty-inducing environment, receivers can receive significantly more offers from proposers when there is less power inequity, but can receive lower offers when there is more power inequity.*

In models 3–5, we considered the dishonest behavior of subjects. These three models only included dishonesty-inducing treatments. We excluded the independent variable dishonest -inducing and added a dummy variable dishonesty, which is equal to 1 if the subject reports fake numbers in Stage 1. We also introduced an interactive variable dishonesty × ultimatum. In these three models, the period positively and significantly impacted the offer (*p* < 0.01). We can see that proposers in the dishonesty-inducing ethical environment would increase their offers to receivers over time. The results also confirm the observed tendency of offers in different treatments in Figure 4. In model 3, self-reported numbers significantly negatively impact the offers to receivers (*p* < 0.01). The subjects who report larger dice numbers provide significantly smaller offers to receivers (*p* < 0.01). Since we found that subjects tell lies to gain power in Stage 1, the larger self-reported numbers have direct relations with dishonesty behavior. We can see that subjects who told greater lies in Stage 1 tend to reduce their offers to receivers.

In models 4 and 5, we considered the impact of proposers’ dishonesty on the money they give to receivers. When considering their interaction, dishonesty significantly negatively impacts the offers to receivers (*p* < 0.01). The two-way interaction term dishonesty × ultimatum also significantly positively impacts offers (*p* < 0.01 These two models indicate that power-holders who use dishonesty to gain power would be more self-interested in Stage 2 and make unfair resource allocation between subjects. However, the interaction term indicates that, in ultimatum games, proposers who report fake numbers in Stage 1 would be more generous in Stage 2. In this ultimatum game design, the receivers hold greater power and dishonest proposers make larger offers to avoid potential rejection from receivers.

**Result** **4.** *Proposers who gain power with dishonest behavior are less generous and behave more self-interestedly in resource allocation. The level of deception can also decrease the intention of providing a larger offer to others. When there is less power inequity between individuals in a group, the dishonesty behavior of power-holders can in turn increase their offers to others.*

In ultimatum game treatments, the subject’s resource allocation behavior is different from the dictator game treatments. The receiver in ultimatum game treatments has veto power, so the power states between subjects are more balanced in the resource allocation. We explored the potential factors that could affect the receiver’s rejection or acceptance actions.

**Figure 5:**
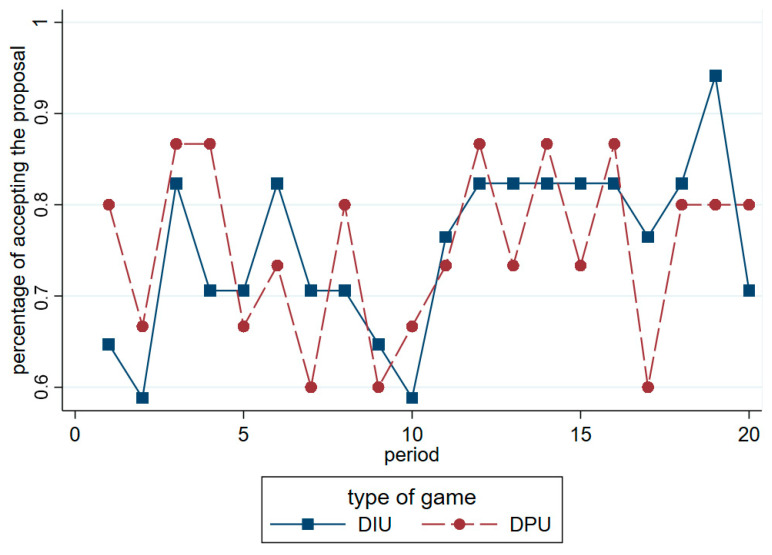
Percentage of receivers’ offer acceptance over time in two ultimatum treatments. Note: DIU represents the dishonesty-inducing ultimatum game; and DPU represents the dishonesty-proof ultimatum game.

First, Figure 5 shows the percentage of receivers’ offer acceptance over time. This descriptive figure shows no differences between the acceptance rates in the two treatments. The results of the Mann–Whitney *U*-test conducted between offer acceptance in the two ultimatum games also shows no significant differences (*z* = −0.016, *p* = 0.987). The dishonesty-inducing or dishonesty-proof treatments are not key variables influencing acceptance. Therefore, we conducted Probit regressions to further analyze the receivers’ acceptance in the two ultimatum game treatments.

Table 4 presents the Probit regression results. In these models, the dependent variable is a dummy variable of offer acceptance, which takes the value 1 if the receiver accepts the offer. Model 1 includes data from all ultimatum game experiment periods. The independent variables in model 1 are: dummy variable dishonesty inducing; period; true dice number; offer; deviations between subjects’ numbers; age; and gender. Coefficients in model 1 indicate that acceptance is directly positively related to the offer, and it is statistically significant (*p* < 0.01). Dishonesty inducing has a significant impact on accepting offers (*p* < 0.01). Receivers would be more likely to accept the offer in the dishonesty-inducing treatments. We compared this result with previous findings in the Tobit regression results. The offer numbers in dishonesty-inducing ultimatum treatments are significantly larger and dishonest power-holders tend to give larger offers. Model 2 only considered the dishonesty-inducing ultimatum treatment. In model 2, when considering subjects’ dishonesty, the results show that receivers significantly increase their rejection actions over time (*p* < 0.05).

**Result** **5.** *In the ultimatum game—in which subjects have more balanced power—receivers have a higher probability of accepting offers, which decreases over time in the dishonesty-inducing treatment.*

## 5. Conclusions

Individuals compete for power in organizations when it can serve their personal interests. In a dishonesty-inducing environment, individuals tend to behave dishonestly to gain power. Our knowledge of the relationship between ethics, deceptive behavior, and the process of gaining and using power remains limited. We examined individuals’ dishonest behavior in different power-balance scenarios and ethical environments. Subjects could experience a sense of inequity in both stages of the experiments: the inequity of power and the inequity of monetary profit. The results showed that subjects in a more balanced power status will be more honest. This could reduce the negative psychological effects of subjects’ dishonest behavior [29]. Subjects might care about financial incentives that concern the punishments from receivers for both their dishonesty and selfishness.

We observed that previous power-holders would have larger intentions of behaving dishonestly to take advantage in order to gain and maintain power. In this study, the two Nash equilibriums of Stage 2 are that if subjects behave on pure self-interest, the proposers should offer nothing or the minimum possible amount. When subjects do not obey the Nash equilibriums and offer more than the minimum amount, it is called altruistic behavior that could reduce moral costs. The offers to others also significantly positively impact their further intentions of behaving dishonestly. Generous power-holders tend to behave more ethically in the future. We observed in our experiment that unethical behavior significantly increases over time in groups. Individuals can learn from the results in previous periods and adjust their dishonest behavior and self-interested behavior in a dishonesty-inducing environment.

Power-holders’ dishonesty has significant negative effects on their offers to receivers. The degree of power-holders’ deception can also significantly negatively affect the offer amounts. Power-holders using unethical methods to gain power are a primary factor that causes inequities in the allocation of collective resources. Our results show that dishonest power-holders with more inequities of power can be less generous and try to maximize personal interest. However, in a more balanced power structure, when receivers hold veto power, power-holders in dishonesty-inducing groups tend to be more generous to others. The interactive factor of dishonest power-holders in the ultimatum treatments significantly positively impacts the offers to receivers. In turn, the receivers tend to accept more in the dishonesty-inducing scenario. However, this mutual benefit action is relatively fragile and the probability of acceptance is negatively impacted by the period.

In our research, we examined how individuals gained and used power in different ethical environments. Our experiments established that power gained through unethical behavior can negatively impact collective interests and produce negative outcomes in the optimization of collective resources for positive organizational aims. From an organizational perspective, we doubt the traditional view that an unbalanced power status causes inequitable distributions of profit. Our results show that when power-holders use unethical methods to gain power, the inequities of organizational resource allocations will be larger and harm collective interests. From a practical perspective, this research suggests that leaders of organizations should avoid the negative impacts of power gained through unethical methods. Collective resource dispersion can negatively influence a workplace climate and workers’ performance and reduce the efficiency and increase invalid competitions in organizations, which could harm the organization’s sustainable value. To prevent disproportionate resource dispersion, managers should create proper ethical environments.

The primary limitation of this research is the small sample size. Another limitation is the fact that the experiments were performed using computers, which may have limited their authenticity. Field experiments should be used in further research to test these relationships under natural conditions. Further studies can also be conducted in cooperative situations to explore ethical behavior in organizations.

## Figures and Tables

**Table 1:** Descriptive analyses results for two experiments.

Descriptive Variables	Dishonesty Inducing		Dishonesty Proof		*t*-Test Result (*p*-Value)
	Mean	Std.	Mean	Std.	
Dice number in dictator game	8.02	2.68	5.43	2.62	−15.64 (*p* *≤* 0.01)
Dice number in ultimatum game	7.44	2.95	5.51	2.66	−12.48 (*p* *≤* 0.01)
Offer in dictator game	2.56	4.67	3.79	5.28	3.91 (*p* *≤* 0.01)
Offer in ultimatum game	10.69	2.74	9.86	3.78	−4.56 (*p* *≤* 0.01)
% of acceptance	0.75		0.75		

**Table 2:** Probit regressions on subject’s dishonesty.

Dependent Variable: Dishonesty	(1)		(2)	
	Coef.	Std. Err.	Coef.	Std. Err.
Ultimatum	−0.511 ***	(0.104)	−0.239 ***	(0.132)
Period	0.026 ***	(0.008)	0.020 ***	(0.008)
True number	−0.160 ***	(0.015)	−0.150 ***	(0.022)
Previous proposer			1.192 ***	(0.090)
Previous offer			−0.043 ***	(0.012)
Previous deviations			0.049 ***	(0.022)
Age	0.004	(0.018)	0.012	(0.018)
Gender	0.331 ***	(0.076)	0.409 ***	(0.092)
Intercept	2.493 ***	(0.428)	2.754 ***	(0.456)
Number of observations	2320		2204	
Pseudo *R*-square	0.100		0.117	

Note: significant codes: *** *p* < 0.01. the robust standard errors are in parentheses on the right.

**Table 3:** Tobit regression on money given to receivers.

Tobit Regression Dependent Variable: Offer Values	(1)	(2)	(3)	(4)	(5)
Dishonesty inducing	−0.165	−2.801 ***			
	(0.212)	(0.612)			
Ultimatum	8.704 ***	7.384 ***	9.816 ***	9.873 ***	8.194 ***
	(0.247)	(0.328)	(0.497)	(0.492)	(0.640)
Period	−0.022	−0.022	0.059 **	0.062 **	0.062 **
	(0.020)	(0.020)	(0.026)	(0.026)	(0.026)
Self-reported number			−0.261 ***	−0.194 ***	−0.203 ***
			(0.049)	(0.066)	(0.065)
Dishonesty				−0.597	−2.796 ***
				(0.396)	(0.809)
Dishonesty inducing × ultimatum		3.652 ***			
		(0.651)			
Dishonesty × ultimatum					3.352 ***
					(0.765)
Gender	−0.761 ***	0.149	−0.411	−0.355	−0.418
	(0.189)	(0.231)	(0.294)	(0.290)	(0.285)
Age	−0.018	−0.008	−0.189 ***	−0.188 ***	−0.183 ***
	(0.046)	(0.046)	(0.066)	(0.066)	(0.065)
Intercept	2.790 ***	2.784 ***	6.590 ***	6.221 ***	7.350 ***
	(1.066)	(1.056)	(1.704)	(1.687)	(1.702)
Number of observations	2320	2320	1120	1120	1120
Pseudo *R*-square	0.10	0.10	0.15	0.15	0.16
*F*-statistic	0.000	0.000	0.000	0.000	0.000

Note: significant codes: *** *p* < 0.01; ** *p* < 0.05. Robust standard errors are in parentheses.

**Table 4:** Probit regression on receivers’ acceptance.

Dependent Variable: Accept Offer	(1)		(2)	
	Coef.	Std. Err.	Coef.	Std. Err.
Dishonesty inducing	0.457 ***	(0.128)		
Period	−0.005	(0.007)	−0.027 **	(0.012)
True dice number	0.004	(0.019)	−0.016	(0.027)
Offer	0.221 ***	(0.016)	0.347 ***	(0.030)
Deviations between subjects’ numbers	0.001	(0.012)	0.015	(0.019)
Dishonesty			−0.165	(0.154)
Age	−0.034	(0.017)	−0.010	(0.031)
Gender	−0.200	(0.089)	−0.119	(0.129)
Intercept	−0.133	(0.433)	0.034	(0.483)
Number of observations	1280		680	
Pseudo *R*-square	0.182		0.270	

Note: significant codes: *** *p* < 0.01; ** *p* < 0.05. Robust standard errors are in parentheses on the right.

## Data Availability

The data used to support the findings of this study are available from the corresponding author upon request.
